# Psychometric properties of the self-report version of the Strengths and Weaknesses of ADHD Symptoms and Normal Behavior Scale in a sample of Hungarian adolescents and young adults

**DOI:** 10.3389/fpsyt.2024.1330716

**Published:** 2024-07-04

**Authors:** Kornél Vajsz, Laura R. Paulina, Salvador Trejo, Adrián A. Andaverde-Vega, James M. Swanson, Mónika Miklósi

**Affiliations:** ^1^ Department of Clinical Psychology, Faculty of Medicine, Semmelweis University, Budapest, Hungary; ^2^ Department of Developmental and Clinical Child Psychology, Psychological Institute, Eötvös Loránd University, Budapest, Hungary; ^3^ Facultad de Medicina y Psicología, Universidad Autónoma de Baja California, Tijuana, Mexico; ^4^ Unidad Académica de Trabajo Social y Ciencias para el Desarrollo Humano, Universidad Autónoma de Tamaulipas, Ciudad Victoria, Mexico; ^5^ Department of Pediatrics, University of California, Irvine, Irvine, CA, United States; ^6^ Centre of Mental Health, Heim Pál National Pediatric Institute, Budapest, Hungary

**Keywords:** ADHD (attention-deficit hyperactivity disorder), adolescence, young Adult, self-report ADHD symptoms, factor structure, Strengths and Weaknesses of ADHD Symptoms and Normal Behavior Scale

## Abstract

The Strengths and Weaknesses of ADHD Symptoms and Normal Behaviour Scale (SWAN) measures the full spectrum of attention and activity symptoms, not just the negative end of the distribution. Previous studies revealed strong psychometric properties of the parent and teacher report versions; however, there is little research on the new self-report form of the SWAN. Therefore, our research aimed to explore the psychometric characteristics of the SWAN self-report. A non-clinical sample of young women (*N* = 664, mean age: 20.01 years, *SD*: 3.08 years) completed the SWAN self-report, the Strengths and Difficulties Questionnaire (SDQ) and the Mental Health Continuum Short Form (MHC-SF). We tested several models using confirmatory factor analyses to assess the factorial validity of the SWAN self-report. Distributional characteristics, convergent, and predictive validity were assessed. A bifactor model with a general factor and a specific inattention factor (bifactor-1) provided the best fit in our data (CFI = 0.977, TLI/NFI = 0.972, RMSEA = 0.053 [90% CI: 0.047 – 0.059], SRMR = 0.061, ω = 0.90). The reliability of the general ADHD factor was good (ω_h_ = 0.87), and the specific inattention factor was acceptable (ω_h_ = 0.73). The distribution of the SWAN self-report scores did not differ from the normal distribution. A strong correlation between the SWAN and the SDQ Hyperactivity subscale was found. The analyses revealed good predictive validity. Our results suggest that the SWAN self-report is a valuable tool for assessing symptoms of ADHD in adolescents and young adults.

## 1 Introduction

Attention-deficit/hyperactivity disorder (ADHD) is a neurodevelopmental disorder characterized by developmentally inappropriate symptoms of inattention, hyperactivity and impulsivity that lead to impairment in at least two domains of functioning (1). According to a recent meta-analysis (2), the prevalence of ADHD is 7.6% in children and 5.6% in adolescents. In adults, it was found to be 2.6% (3), with a risk as high as 70–75% for coexisting psychiatric disorders (4, 5), including mood and anxiety disorders, disruptive disorders, bipolar disorder, substance use, behavioural addictions, insomnia, and personality disorders (6–13). Though many environmental and genetic factors with small effects have already been identified, there are no definitive causal factors that can reliably predict ADHD (14–17). In their highly important work, Kapteyn and van Uven (18) proved that a high number of independent factors of small effects tend to cause normal distributions, whereas processes with few factors and large effects tend to cause more skewed distributions. In line with this, ADHD-related traits follow a continuous, normal distribution in the population (19, 20).

In children, the diagnosis is mainly based on parent and teacher ratings, as well as on the direct observation of the symptoms (21). The importance of self-reporting increases with age, however. Though parent-report predicts outcomes better than self-reports even in adolescents and college students (22, 23), by adulthood, self-report is the most valuable source of information in the diagnostic process (24). It should be noted that symptom list-based approaches are the predominant means of assessing adult ADHD through rating scales, such as the Adult AD/HD Rating Scale [AARS (25)], the Barkley Adult ADHD Rating Scale–IV [BAARS-IV (26)], the Conners’ Adult ADHD Rating Scale [CAARS (27)], and the Adult ADHD Self-Report Scale [ASRS (28)]. Although these measures have strong psychometric properties, they indicate normative functioning by the absence of symptoms, resulting in a skewed and truncated distribution of scores. The lack of fit to normal distribution causes difficulty in establishing the clinical cutoffs of symptom scales, which can lead to lower specificity and type 1 errors (29). Therefore, it is important to take the strengths in attention, activity, and impulse control into consideration as well to see the full picture (19, 20).

The Strengths and Weaknesses of ADHD Symptoms and Normal Behaviour Scale [SWAN (20)] was shown to satisfy this requirement. SWAN is an 18-item questionnaire with a 7-point Likert scale that examines traits related to ADHD symptoms. The scale is anchored to average behaviour, namely that –3 means the respondent observed far below average capability in the trait measured, 0 means average behaviour, and +3 means far above average ability that can be a strength in self-control or executive functioning (i.e., *lower* scores indicate *greater* difficulties). Consequently, the SWAN scores were shown to follow a normal distribution (20, 30–32), and, therefore, it was found to have just as good sensitivity but better specificity than other ADHD symptom scales (33). Originally, parent-, and teacher-report versions have been developed (20), and translated into several languages (30, 31, 33–37). More recently, Blume and colleagues (38) created a self-report version the German language, extending the SWAN family.

The Diagnostic and Statistical Manual of Mental Disorders, Fifth Edition [DSM-5 (1)] conceptualizes ADHD with two primary dimensions; consequently, predominantly inattentive, predominantly hyperactive/impulsive, and combined presentations are described. However, it has been debated whether the two-factor structure best represents ADHD (39). Factor-analytic studies have reported the best fit for different measurement models, with mixed support for one-, two-, and three-factor models or more complex hierarchical or bifactor models. In terms of fit indices, most evidence seems to support the bifactor models (40–43). In bifactor models, a latent general ADHD construct may explain the diagnostic stability of ADHD, while the specific factors are in line with the clinical observation that symptom presentation may change over time (44). Converging results revealed a strong and reliable general ADHD factor in different age groups and across different measures (40). A specific inattention factor which acquired sufficient specificity and stability for interpretation beyond the general ADHD factor has also been supported by several studies, especially in clinical samples (42). However, a specific hyperactivity/impulsivity factor (bifactor-2 model) or separate hyperactivity and impulsivity factors (bifactor-3 models) were shown to explain only a small amount of additional variance and had low reliabilities and low construct replicabilities. It is important to note that, bifactor-3 models performed better when *talks excessively* (modulating verbal activity) was treated as a part of impulsivity rather than hyperactivity (41, 43). In line with this several authors argued that the hyperactivity and impulsivity symptoms listed in the DSM-5 (1) could be better grouped into motor and verbal hyperactivity/impulsivity (45). The weakness of specific factors led some researchers to view bifactor models as not interpretable, clinically irrelevant, or even statistical artefacts (46, 47). In that way, they suggested the use of either a total score of the scales (the general ADHD factor only) (46) or the acceptance of more traditional non-hierarchical two- or three-factor models (47) or hierarchical models (39), despite of their inferiority to bifactor models in term of fit indices. However, the hierarchical models had negative factor loadings and low internal consistencies which is a known issue for models with a general factor (48). The usage of bifactor-1 model was found to be a viable alternative by previous ADHD researchers (49, 50).

The factor structure of the SWAN has also been explored. In studies on the parent- and teacher-report versions, exploratory factor analysis resulted in a two-factor structure (inattention and hyperactivity/impulsivity) in different samples (20, 31). In studies using confirmatory factor analysis (31, 35, 51, 52), bifactor models were reported to have a better fit than non-hierarchical models; however, similar to studies with other measures, the results suggested a strong and reliable general ADHD factor but mostly weak or unreliable specific factors. A single study under peer review (38) reported similar results for the new self-report version of the SWAN. Blume et al. found an inferior fit of a non-hierarchical two-factor model in comparison to a bifactor model with a general and two specific factors; however, five of nine item-loadings of the specific hyperactivity/impulsivity factor were nonsignificant.

To gain more information about the self-report version of the SWAN, our study aimed to examine the psychometric properties of the Hungarian version in a sample of adolescents and young adults.

## 2 Methods

### 2.1 Sample and procedures

The Institutional Research Ethics Committee of the Psychological Institute Eötvös Loránd University approved the study (Nr. 2022/344). There was a 24-hour-long campaign through Mélylevegő Projekt (Deep Breath Project), a Hungarian psychoeducational social media page. Mélylevegő Projekt was founded during the 2020 COVID-19 lockdown by graduate psychology students as a platform connect and provide mental health related information for teens and young adults. Participants gave their informed consent before completing the online questionnaire.

In total, 2597 participants agreed to fill out the questionnaire, of whom 717 completed it. Completers’ gender distribution was highly skewed (*N* = 664 [92.6%] women, *N* = 47 [6.6%] men, *N* = 6 [0.8%] other), therefore, data from 664 women (age range: 14 − 25 years, mean age: 20.01 years, *SD*: 3.08) were analyzed (**Table 1**).

**Table 1:** Demographic characteristics (*N* = 664).

Demographics	*N (%)*
Subsample	
Adolescents (14 - 18 years)	178 (26.8)
Adult (> 18 years)	486 (73.2)
Economic activity	
Student	557 (83.9)
Primary school	14 (2.1)
Vocational secondary school	51 (9.0)
High school	174 (26.2)
Undergraduate student	216 (32.5)
Graduate student	70 (10.5)
Postgraduate student	20 (3.0)
Internship	5 (0.8)
Economically active	83 (12.5)
Unemployed	14 (2.1)
Location of residence	
Capital	164 (24.7)
Urban areas	330 (49.7)
Rural areal	169 (25.5)
Financial status	
Below average	33 (5.0)
Average	518 (78.0)
Above average	113 (17.0)

### 2.2 Measure

The self-report version of the SWAN (38) was used to assess ADHD symptoms. Blume et al. (38) reported good internal reliabilities for the German version (α = 0.85 – 0.90). Up to now, only the parent and teacher versions of the SWAN have been validated in the Hungarian language showing good internal consistencies (α = 0.87 – 0.93) and convergent validity with the Hyperactivity subscale of the Strengths and Difficulties Questionnaire [SDQ (53)] (*r* = 0.67 - 0.74) (30). The Hungarian version of the SWAN self-report was created by Paulina et al. (54). In their pilot study, they reported good internal reliability (α = 0.89) for the total score, but the factorial validity has not been evaluated yet.

The SDQ (53) assesses emotional and behavioral problems with four problem scales (Emotional problems, Conduct problems, Hyperactivity, Peer problems) and prosocial skills. The SDQ Hyperactivity subscale measures hyperactivity with two items (SDQ#2, SDQ#10), but includes an impulsivity item (SDQ#20) and two inattention items (SDQ#15, SDQ#25) as well. Higher scores indicate more emotional and behavioral problems. Internal reliabilities of the self-report version were α_SDQ-ES_ = 0.66, α_SDQ-CP_ = 0.60, α_SDQ-H_ = 0.67, α_SDQ-PP_ = 0.41, α_SDQ-P_ = 0.66 (53). Psychometric properties of the Hungarian version have been reported by Turi et al. (55). In our sample, the Hyperactivity and Emotional problems subscales had acceptable reliability (α_SDQ-H_ = 0.76 and α_SDQ-ES_ = 0.68), but the Conduct problems and Peer problems subscales had poor reliabilities (α_SDQ-CP_ = 0.47, α_SDQ-PP_ = 0.45), therefore, we used only the Hyperactivity and Emotional problems subscales for further analyses.

The Mental Health Continuum - Short Form [MHC-SF (56)] measures emotional, psychological and social well-being with 14 Likert-type items measuring the frequency of indicators of positive mental health (from 1 = never to 6 = every day). Both the original (α_MHC_ = 0.74) and the Hungarian versions (α_MHC_ = 0.89) showed good internal consistencies (56, 57). In the present sample, the internal reliability was α_MHC_ = 0.85.

### 2.3 Statistical analyses

Confirmatory factor analyses were conducted to assess the factorial validity of the SWAN self-report. Because of the significance of the Shapiro-Wilk Test for Multivariate Normality and the Mardia’s coefficients for multivariate skewness and kurtosis, we used the Diagonally Weighted Least Squares (DWLS) estimator (58). We compared several models: a non-hierarchical two-factor model and a bifactor model with a general ADHD factor and two specific factors for inattention and hyperactivity/impulsivity (bifactor-2), a bifactor model with a general factor and a specific inattention factor (bifactor-1), and two bifactor models with a general and three specific factors: inattention, hyperactivity + “Modulating verbal activity”, and impulsivity (bifactor-3-IA/H[MVA]/I), and inattention, hyperactivity, and hyperactivity+ “Modulating verbal activity” (bifactor-3-IA/H/I[MVA]). The analyses were conducted using the *lavaan* (version 0.6–15) package of JASP version 0.17.1 and R version 4.2.1. Factor scaling was assumed to be based on factor variances. Maximum likelihood estimators were used. Comparative Fit Indices (CFI) and Tucker-Lewis Indices/Normed Fit Indices (TLI/NFI) were calculated with a minimum acceptable threshold of 0.900 (59, 60). Root Mean Square Error of Approximation (RMSEA) was calculated with a maximum acceptable threshold of 0.07 (61). Standardized Root Mean Square Residual (SRMR) was calculated with a maximum acceptable threshold of 0.08 (62). Composite reliabilities were calculated with ω_h_ > 0.50 as the criteria of minimal acceptability and ω_h_ > 0.75 as the preferred reliability index (63). Explained common variance was calculated for the bifactor models to compare the factor loadings of the general factor with that of the one-factor model (64). Hancock’s H coefficient was calculated for each factor to measure how well each latent variable is represented by its group of items. The criterium of stability was H > 0.70, which means the factor is unlikely to fluctuate across samples (47, 65).

Normality was evaluated by the Shapiro-Wilk statistics, skewness, and kurtosis. Convergent validity was evaluated by computing the Person’s correlational coefficient of the SWAN and the SDQ Hyperactivity subscale. Predictive validity was examined by means of linear regression analyses with the MHC-SF and the SDQ Emotional problems subscale as dependent variables.

## 3 Results

### 3.1 Confirmatory factor analyses

First, the non-hierarchical two-factor model was evaluated. The usage of Chi-square goodness of fit was largely eschewed due to the large sample size (66). Composite reliability of the two-factor model was ω_h_ = 0.85 for inattention, ω_h_ = 0.87 for hyperactivity/impulsivity, and ω = 0.92 for the total model. Construct replicability was good for both inattention (Hancock’s H-index = 0.86) and hyperactivity/impulsivity (Hancock’s H-index = 0.87) factors. The model showed a good fit to our data (CFI = 0.972, TLI/NFI = 0.968, RMSEA = 0.057 [90% CI: 0.051 – 0.063], SRMR = 0.066). All item loadings were significant (**Table 2A**).

**Table 2A:** Factor loadings and fit indices of the two-factor, bifactor-2, and bifactor-1 models.

Item	Model									
	Two-factor			Bifactor-2				Bifactor-1		
	SWAN-IA	SWAN-HI	*R^2^*	SWAN-IA	SWAN-HI	SWAN Σ	*R^2^*	SWAN-IA	SWAN Σ	*R^2^*
1 Attending to detail	0.463		0.215	0.383		0.282	0.226	0.384	0.281	0.227
2 Sustaining attention	0.704		0.496	0.651		0.392	0.577	0.649	0.395	0.578
3 Listening	0.676		0.457	0.362		0.514	0.394	0.358	0.516	0.395
4 Following through	0.709		0.503	0.540		0.449	0.493	0.537	0.453	0.493
5 Organizing	0.538		0.289	0.530		0.280	0.359	0.528	0.283	0.359
6 Engaging in sustained effort	0.547		0.299	0.494		0.310	0.340	0.496	0.308	0.341
7 Keeping track of things	0.681		0.464	0.531		0.422	0.459	0.529	0.424	0.459
8 Ignoring extraneous stimuli	0.512		0.262	0.325		0.359	0.234	0.322	0.362	0.235
9 Remembering	0.682		0.466	0.512		0.434	0.450	0.508	0.438	0.450
10 Sitting still		0.645	0.416		0.437	0.612	0.566		0.644	0.415
11 Staying seated		0.678	0.460		0.419	0.647	0.594		0.678	0.460
12 Modulating motor activity		0.671	0.450		0.468	0.642	0.631		0.671	0.450
13 Appropriate noise level		0.608	0.369		0.159	0.583	0.365		0.606	0.367
14 Settling down		0.744	0.554		0.442	0.711	0.701		0.745	0.555
15 Modulating verbal activity		0.680	0.463		–0.264	0.736	0.611		0.679	0.461
16 Reflecting on questions		0.602	0.363		–0.354	0.680	0.587		0.602	0.363
17 Awaiting turn		0.634	0.401		–0.147	0.658	0.455		0.634	0.402
18 Entering into conversations		0.558	0.311		–0.213	0.596	0.401		0.559	0.313
df	134			117				126		
χ^2^ (*p*)	419.080 (< 0.001)			131.127 (0.176)				360.413 (< 0.001)		
Comparative Fit Index (CFI)	0.972			0.999				0.977		
Tucker-Lewis Index (TLI/NFI)	0.968			0.998				0.972		
RMSEA [90% CI]	0.057 [0.051 – 0.063]			0.013 [0.000 – 0.024]				0.053 [0.047 – 0.059]		
SRMR	0.066			0.036				0.061		
Composite reliability (ω)	0.92			0.89						0.90
Composite reliability (ω_h_)	0.85	0.87		0.73	0.10	0.87		0.73	0.87	
Hancock’s H-index	0.86	0.87		0.75	0.56	0.90		0.75	0.89	
Explained Common Variance				0.62						0.71

N = 664. SWAN, Strength and Weaknesses of ADHD Symptoms and Normal Behaviour Scale; SWAN Σ, SWAN total score; SWAN-IA, SWAN Inattention factor; SWAN-HI, SWAN Hyperactivity/Impulsivity factor; SWAN-M, SWAN Motor hyperactivity/impulsivity factor; SWAN-V, SWAN Verbal hyperactivity/impulsivity factor.

The bifactor-2 model showed a very good fit (CFI = 0.999, TLI/NFI = 0.998, RMSEA = 0.013 [90% CI: 0.000 – 0.024], SRMR = 0.036), and the SRMR has shown a considerable decrease compared to the two-factor model (ΔSRMR = 0.030). All item loadings were statistically significant. Items 15–18 demonstrated significant, negative loadings on the hyperactivity/impulsivity factor. The overall reliability of the bifactor-2 model was good (ω = 0.89). The reliability of the general ADHD factor was also good (ω_h_ = 0.87), the specific inattention factor was minimally acceptable (ω_h_ = 0.73), and the reliability of the specific hyperactivity/impulsivity factor was poor (ω_h_ = 0.10). The explained common variance of the model was ECV = 0.62, suggesting that the factor loadings of the general ADHD factor were slightly similar to those that might be obtained with the one-factor model. Analysis of construct replicability indicated that the general ADHD factor (Hancock’s H-index = 0.90) and the specific inattention factor (Hancock’s H-index = 0.76) were stable, but the specific hyperactivity/impulsivity factor was not (Hancock’s H-index = 0.56).

The bifactor-1 model showed a good fit (CFI = 0.977, TLI/NFI = 0.972, RMSEA = 0.053 [90% CI: 0.047 – 0.059], SRMR = 0.061). All factor loadings were significant, ranging from 0.28 to 0.75. The factor loadings of six of nine inattention-symptom items were substantially larger than their loadings on the general factor (Δ*b* > 0.1). The overall reliability of the bifactor-1 model was good (ω = 0.90). The reliability of the general ADHD factor was also good (ω_h_ = 0.87), and the specific inattention factor was minimally acceptable (ω_h_ = 0.73). The explained common variance was 0.71, suggesting that the general ADHD factor explained more than two-thirds of the common variance. Analysis of construct replicability indicated that both the general ADHD factor (Hancock’s H-index = 0.89) and the inattention factor (Hancock’s H-index = 0.75) were stable.

Both bifactor-3 models (**Table 2B**) showed very good fit [bifactor-3-IA/H[MVA]/I: CFI = 0.995, TLI/NFI = 0.994, RMSEA = 0.025 [90% CI: 0.015 – 0.033], SRMR = 0.041; bifactor-3-IA/H/I[MVA]: CFI = 0.998, TLI/NFI = 0.998, RMSEA = 0.015 [90% CI: 0.000 – 0.025], SRMR = 0.037)], with the bifactor-3-IA/H/I[MVA] performing slightly better. “Modulating verbal activity” had a negative loading on the Hyperactivity factor (bifactor-3-IA/H[MVA]/I model) but loaded significantly and positively on the Impulsivity factor (bifactor-3-IA/H/I[MVA] model). In both models, the overall reliability of the model was good (ω = 0.91 for both); the reliability of the general ADHD factor was also good (ω_h_ = 0.86 for both), and the specific inattention factor was minimally acceptable (ω_h_ = 0.70 and 0.68, respectively). However, in both bifactor-3 models, the reliabilities of the hyperactivity and impulsivity factors were poor (< 0.70). The explained common variances of the models were 0.58 and 0.56, respectively, suggesting the general ADHD factor explained about half of the common variance in the bifactor-3 models. Construct replicability indicated that, both the general ADHD factor (Hancock’s H-index = 0.88 and 0.87, respectively) and the inattention factor (Hancock’s H-index = 0.73 and 0.71, respectively) were stable in both bifactor-3 models. The hyperactivity factor was unstable in both models (Hancock’s H-index = 0.65); however, the impulsivity factor did not show stability in both bifactor-3 models (Hancock’s H-index = 0.27 and 0.49, respectively).

**Table 2B:** Factor loadings and fit indices of t of the bifactor-3 models.

Item	Model									
	Bifactor-3-IA/H[MVA]/I					Bifactor-3-IA/H/I[MVA]				
	SWAN-IA	SWAN-H	SWAN-I	SWAN Σ	R^2^	SWAN-IA	SWAN-M	SWAN-V	SWAN Σ	R^2^
1 Attending to detail	0.368			0.306	0.229	0.363			0.315	0.231
2 Sustaining attention	0.631			0.427	0.580	0.617			0.448	0.581
3 Listening	0.309			0.558	0.406	0.273			0.584	0.415
4 Following through	0.506			0.488	0.494	0.484			0.510	0.495
5 Organizing	0.523			0.304	0.365	0.519			0.317	0.370
6 Engaging in sustained effort	0.477			0.338	0.341	0.469			0.351	0.343
7 Keeping track of things	0.494			0.461	0.456	0.468			0.484	0.453
8 Ignoring extraneous stimuli	0.290			0.390	0.236	0.266			0.409	0.238
9 Remembering	0.476			0.472	0.449	0.450			0.495	0.448
10 Sitting still		0.460		0.545	0.509		0.443		0.552	0.500
11 Staying seated		0.543		0.562	0.610		0.553		0.557	0.616
12 Modulating motor activity		0.611		0.544	0.670		0.623		0.537	0.677
13 Appropriate noise level		0.262		0.545	0.365		0.273		0.540	0.366
14 Settling down		0.576		0.622	0.718		0.578		0.617	0.715
15 Modulating verbal activity		–0.101		0.751	0.574			0.446	0.647	0.618
16 Reflecting on questions			0.320	0.611	0.476			0.594	0.558	0.665
17 Awaiting turn			0.360	0.631	0.527			0.264	0.616	0.449
18 Entering into conversations			0.312	0.559	0.410			0.280	0.540	0.370
df	117					117				
χ^2^ (*p*)	165.327 (0.002)					133.312 (0.144)				
Comparative Fit Index (CFI)	0.995					0.998				
Tucker-Lewis Index (TLI/NFI)	0.994					0.998				
RMSEA [90% CI]	0.025 [0.015 – 0.033]					0.015 [0.000 – 0.025]				
SRMR	0.041					0.037				
Composite reliability (ω)	0.91					0.91				
Composite reliability (ω_h_)	0.70	0.62	0.18	0.86		0.68	0.62	0.43	0.86	
Hancock’s H-index	0.73	0.65	0.27	0.88		0.71	0.65	0.49	0.87	
Explained Common Variance	0.58					0.56				

N = 664. SWAN, Strength and Weaknesses of ADHD Symptoms and Normal Behaviour Scale; SWAN Σ, SWAN total score; SWAN-IA, SWAN Inattention factor; SWAN-HI, SWAN Hyperactivity/Impulsivity factor; SWAN-M, SWAN Motor hyperactivity/impulsivity factor; SWAN-V, SWAN Verbal hyperactivity/impulsivity factor.

We compared the models by computing several Scaled Chi-Square Difference Tests. All bifactor models were proven to be significantly better than the two-factor model. However, the bifactor-2 (Δχ^2^(9) = 229.286, p < 0.05), and both the bifactor-3-IA/H[MVA]/I (Δχ^2^(9) = 195.086, p < 0.05) and bifactor-3-IA/H/I[MVA] models (Δχ^2^(9) = 227.101, p < 0.05) were significantly better than the bifactor-1 model.

### 3.2 Distributional characteristics of the SWAN

Shapiro-Wilk statistics were non-significant for both adolescents (*W* = 0.994, *p* = 0.700) and young adults (*W* = 0.995, *p* = 0.137), indicating normality. The skewness of the SWAN scores in the adolescent and young adult samples was found to be 0.03 and 0.02, respectively, indicating a close fit to symmetrical distribution. The kurtosis of SWAN in the adolescent and young adult samples was found to be –0.39 and –0.44, respectively.

### 3.3 Convergent validity

Descriptive statistics and bivariate relationships of study variables are shown in **Table 3**. The SWAN subscales showed significant negative correlations of large effect size with the SDQ Hyperactivity subscale indicating good convergent validity (**Table 3**).

**Table 3:** Descriptive statistics, reliabilities and intercorrelations of study variables (Pearson’s correlational coefficients).

	α[95% CI]	Mean	SD	SWAN Σ	SWAN-IA	SWAN-HI	SDQ-ADHD	SDQ-EMO
SWAN Σ	0.89 [0.88 – 0.91]	0.267	16.322					
SWAN-IA	0.84 [0.82 – 0.86]	–0.002	9.176	0.873				
SWAN-HI	0.86 [0.85 – 0.88]	0.268	9.444	0.880	0.537			
SDQ-ADHD	0.76 [0.73 – 0.79]	6.556	2.320	–0.756	–0.639	–0.686		
SDQ-EMO	0.68 [0.64 – 0.72]	4.723	2.521	–0.366	–0.346	–0.297	0.365	
MHC-SF	0.85 [0.83 – 0.86]	48.361	11.015	0.356	0.364	0.262	–0.283	–0.449

N = 664. SWAN, Strength and Weaknesses of ADHD Symptoms and Normal Behavior Scale, self-report version; SDQ, Strengths and Difficulties Questionnaire; SWAN Σ, SWAN self-report total score; SWAN-IA, SWAN self-report Inattention; SWAN-HI, SWAN self-report Hyperactivity/Impulsivity. SDQ-EMO, SDQ Emotional Problems; SDQ-ADHD, SDQ Hyperactivity; MHC-SF, Mental Health Continuum Short Form. All correlational coefficients were significant at the p < 0.001 level.

### 3.4 Predictive validity

In a linear regression model with MHC-SF as the dependent variable and the total score of the SWAN self-report scale as an independent variable, the analysis resulted in a significant model (adjusted *R^2^* = 0.125, *F*(1,663) = 96.125, *p* < 0.001). The SWAN was a significant predictor (β = 0.356, *p* < 0.001), explaining 12.5% of the variance of the dependent variable. When choosing the SDQ Hyperactivity subscale as the independent variable, the regression model was significant (adjusted *R^2^* = 0.079, *F*(1,662) = 57.486, *p* < 0.001). The SDQ Hyperactivity was a significant predictor (β = -0.283, *p* < 0.001), explaining 7.9% of the variance of the dependent variable.

The next model, with SDQ Emotional problems as dependent and the SWAN as the independent variable, was significant (adjusted *R^2^* = 0.133, *F*(1,662) = 102.694, *p* < 0.001). The SWAN was a significant predictor (β = –0.366, *p* < 0.001). Finally, the regression analysis with SDQ Emotional problems as dependent and the SDQ Hyperactivity scale as an independent variable resulted in a significant model (adjusted *R^2^* = 0.132, *F*(1,662) = 101.770, *p* < 0.001). The SDQ Hyperactivity was a significant predictor (β = 0.365, *p* < 0.001).

## 4 Discussion

There is a solid theoretical foundation for the relevance of measuring the full spectrum of ADHD symptoms, not just the negative end of the distribution (19, 20, 32). Also, several empirical studies using parent or teacher-report versions of the SWAN scale have demonstrated the merits of this approach [e.g (30, 67). (31, 34, 33) (35–37)]. Up to now, there was no self-report instrument available that would measure both strengths and weaknesses in attention, activity, and impulse control. Blume and colleagues (38) have filled this gap by developing a self-report version of the SWAN. Since they reported promisingly good psychometric characteristics for the German version of the SWAN self-report, it was worthwhile to conduct further research with the Hungarian version. Therefore, our research aimed to explore the factor structure, and distributional characteristics of the SWAN self-report, and to assess its convergent validity with the SDQ Hyperactivity subscale. Furthermore, we assessed its predictive validity in linear regression models by analyzing how well it can explain the variance in mental well-being and emotional problems.

### 4.1 The factor structure of the SWAN self-report

Though all models tested showed acceptable fit, the bifactor models were superior to the non-hierarchical two-factor model in multiple fit indices: they had higher CFI and NFI/TLI and lower 90%CI for the RMSEA and SRMR. However, though the bifactor-2 and bifactor-3 models have shown a better fit than the bifactor-1, neither has shown an acceptable composite reliability nor Hancock’s H-index for each of their factors. The specific hyperactivity/impulsivity factor of the bifactor-2 model was shown to have poor composite reliability of ωh = 0.10. The items of the specific factor thus share 1% in common variance, the rest being accountable for measurement error. The bifactor-3 models were also shown to have poor composite reliabilities. Bifactor-3-IA/H[MVA]/I’s specific impulsivity factor was ωh = 0.18, having its items share 3.2% in common variance. Bifactor-3-IA/H/I[MVA]’s specific verbal hyperactivity/impulsivity factor was ωh = 0.43 having its items share 18.5% in common variance. The model with the best fit to show good composite reliability was the bifactor-1 model.

When evaluating the different factors, we concluded that both the general ADHD factor and the specific inattention factor had acceptable composite replicability and stability in the bifactor models. Our results suggested that more than two-thirds of the variance was explained by a single common source, i.e., a general ADHD factor, and there was also a substantial proportion of unique variance associated with the inattention factor. However, after partitioning out the variability explained by the general factor, there was little variance remaining that could have been attributed to the (joint or separated) specific hyperactivity and impulsivity factors. The comparison of the bifactor-3 models revealed that “modulating verbal activity” fitted better to the impulsivity than the hyperactivity factor, as suggested in previous research (41).

These findings converge with the results of previous studies exploring the structure of ADHD symptoms in different age groups and across different measures (40, 52, 68) and are in line with the findings of the single study on the SWAN self-report (38). On the other hand, diverging interpretations of similar findings have been formulated by different researchers. Some concluded that the bifactor models support the unidimensionality of ADHD symptoms and suggested using the total score (46). In contrast, Park and colleagues (47) recalled research findings showing different associations of attention deficit and hyperactivity/impulsivity factors with comorbid conditions, cognitive variables, and different domains of functional impairment (69), underlining the importance of different ADHD domains. They have argued that bifactor models only perform better because of their complexity, and in this way, they can be considered statistical artefacts and are not clinically useful. Our results support the notion that the only use of a general ADHD factor would lead to missing important information about the heterogeneity of the phenomenon; the inclusion of a separate inattention factor in the model is at least warranted. On the other hand, similarly to previous findings, our analysis found the hyperactivity and impulsivity factors to be problematic.

On one hand, these results may be related to the questionable validity or at least the low prevalence of predominantly hyperactive/impulsive presentation of ADHD, especially in adolescents and adults when hyperactivity typically decreases (70). For example, in a large sample of adults with ADHD, the prevalence of a clinical diagnosis of ADHD with hyperactive/impulsive presentation was very rare (3%), and this group had ratings of inattention comparable to the group with a clinical diagnosis of ADHD inattentive presentation (71). Furthermore, latent class analysis did not reveal a group with predominantly hyperactivity and impulsivity in children and adolescents aged between 6 and 18 years old (72). These results suggest that hyperactivity/impulsivity symptoms are accompanied by inattention symptoms in the vast majority of the cases, in that way, in factor-analytic studies they would be captured by the general ADHD factor.

On the other hand, DSM-based rating scales follow a relatively simplistic conceptualization of impulsivity as a single domain, however, impulsivity has been defined as a multidimensional construct in previous research (73, 74). Furthermore, all but one symptom of impulsivity in the DSM-5 symptom list (75) is specifically related to the verbal modality, while the assessment of motor impulsivity is very limited. These shortcomings may prevent the definition of an interpretable impulsivity factor in studies using these rating scales.

### 4.2 The distributional characteristics and the validity of the SWAN self-report

In line with previous research using different versions of the SWAN (30, 38), we found that the distribution of the SWAN self-report scores did not differ significantly from the normal distribution. Our analysis also confirmed the good convergent validity of the SWAN self-report by its very strong correlation with the SDQ Hyperactivity subscale. The correlation was even higher than the correlation of SWAN self-report with the Conners’ Adult ADHD Rating Scale (38) and that of the parent-report versions of the SWAN and the SDQ Hyperactivity subscale (34) in previous research.

Both measures explained a significant proportion of variance in emotional problems and mental health. In the case of the MHC-SF, the predictive power of the SWAN was better than that of the SDQ Hyperactivity. In previous research using the parent-report version, the association of the SWAN and SDQ Emotional problems was somewhat lower than in our study (34), which may be due to the different informants used.

### 4.3 Limitations

The results can be viewed considering the limitations of the study. The cross-sectional design does not allow us to explore causality. Self-reports of ADHD symptoms and mental health may be biased by social desirability, insight, and cognitive abilities. Our sample consisted solely of women which may be related to the data collection method; young women may be more interested in mental health topics covered by the Mélylevegő Project website than men. Therefore, our results cannot be generalized to the population, future research should address these psychometric properties in more diverse demographic groups. Gender differences have been reported in symptom presentation, prevalence, comorbid profile, and social perception of ADHD symptoms (76, 77). Symptoms of inattention are more likely to be present than hyperactive symptoms in women, sometimes leading to delayed referral and diagnosis (77, 78). However, factor analytic studies reported gender invariance in adolescent (40) and adult (35) samples, suggesting that gender differences might not have a substantial influence on the results. On the other hand, cultural differences have been reported in the structure of ADHD symptoms; more research is needed in different cultural contexts (79).

## 5 Conclusion

Taken together, our results suggest that the SWAN self-report is a valuable tool for assessing symptoms of ADHD in adolescents and young adults. It has comparably good psychometric properties to measures in the SWAN family.

## Data availability statement

The datasets presented in this study can be found in online repositories. The names of the repository/repositories and accession number(s) can be found below: https://osf.io/frvsw/?view_only=b9e81e7a8d824881b32070984b2a0ae6.

## Ethics statement

The Institutional Research Ethics Committee of the Psychological Institute Eötvös Loránd University approved the study (Nr. 2022/344). The studies were conducted in accordance with the local legislation and institutional requirements. Written informed consent for participation in this study was provided by the participants’ legal guardians/next of kin.

## Author contributions

KV: Conceptualization, Formal analysis, Funding acquisition, Investigation, Methodology, Project administration, Software, Visualization, Writing – original draft, Writing – review & editing. LP: Conceptualization, Methodology, Writing – review & editing. ST: Methodology, Writing – review & editing, Supervision. AA-V: Methodology, Supervision, Writing – review & editing. JS: Methodology, Supervision, Writing – review & editing. MM: Methodology, Supervision, Writing – review & editing, Conceptualization, Formal analysis, Writing – original draft.
