# Supplementary figures and images for: Psychometric properties of the self-report version of the Strengths and Weaknesses of ADHD Symptoms and Normal Behavior Scale in a sample of Hungarian adolescents and young adults
Source: Front Psychiatry. 2024 Jul 4;15:1330716. doi: 10.3389/fpsyt.2024.1330716 (PMC11255780; doi:10.3389/fpsyt.2024.1330716)

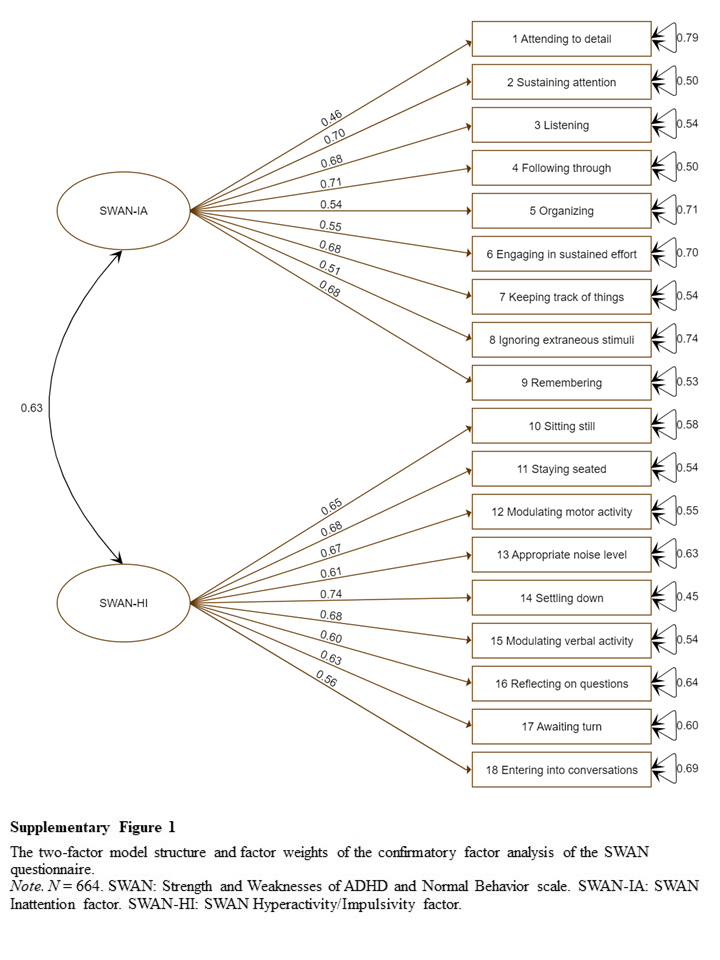

Supplement: Supplementary file 1 [file DataSheet_1.zip › Supplementary Figure 1.TIF]

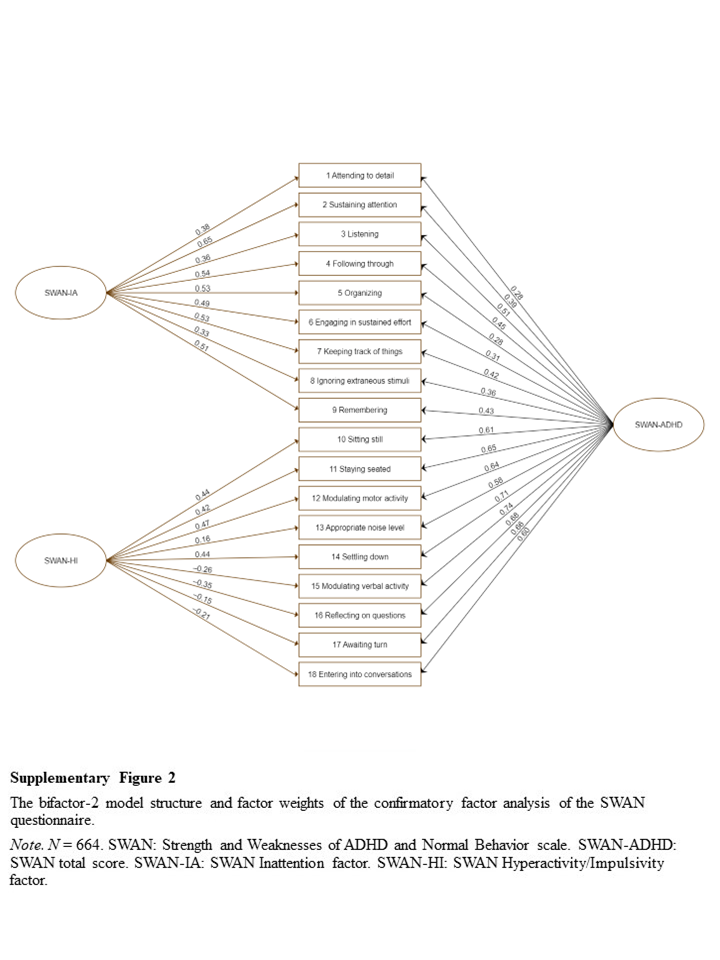

Supplement: Supplementary file 1 [file DataSheet_1.zip › Supplementary Figure 2.TIF]

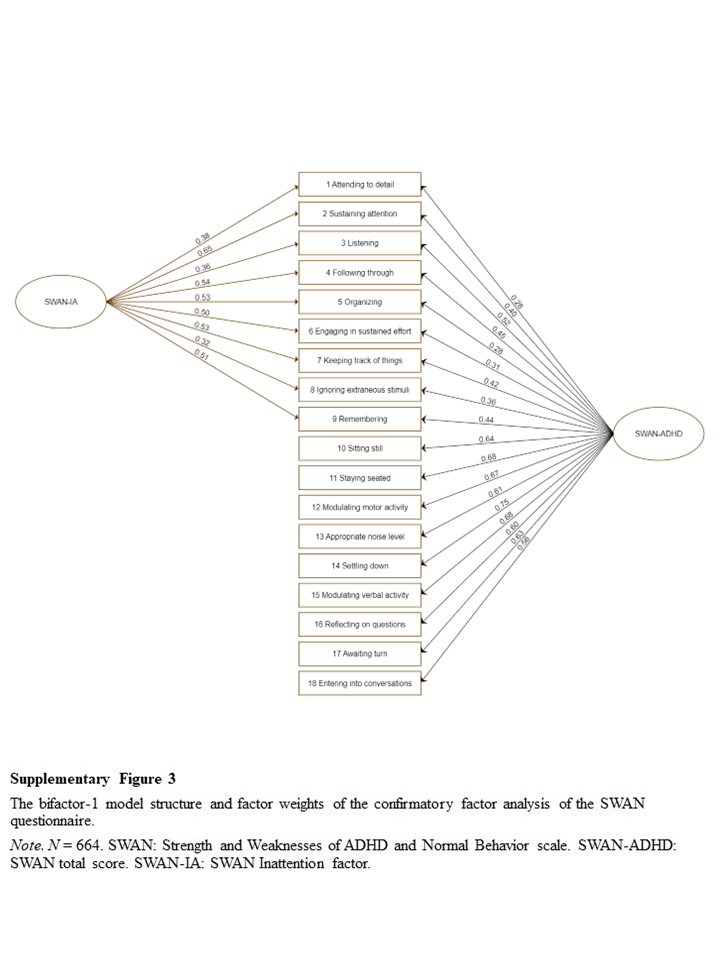

Supplement: Supplementary file 1 [file DataSheet_1.zip › Supplementary Figure 3.TIF]

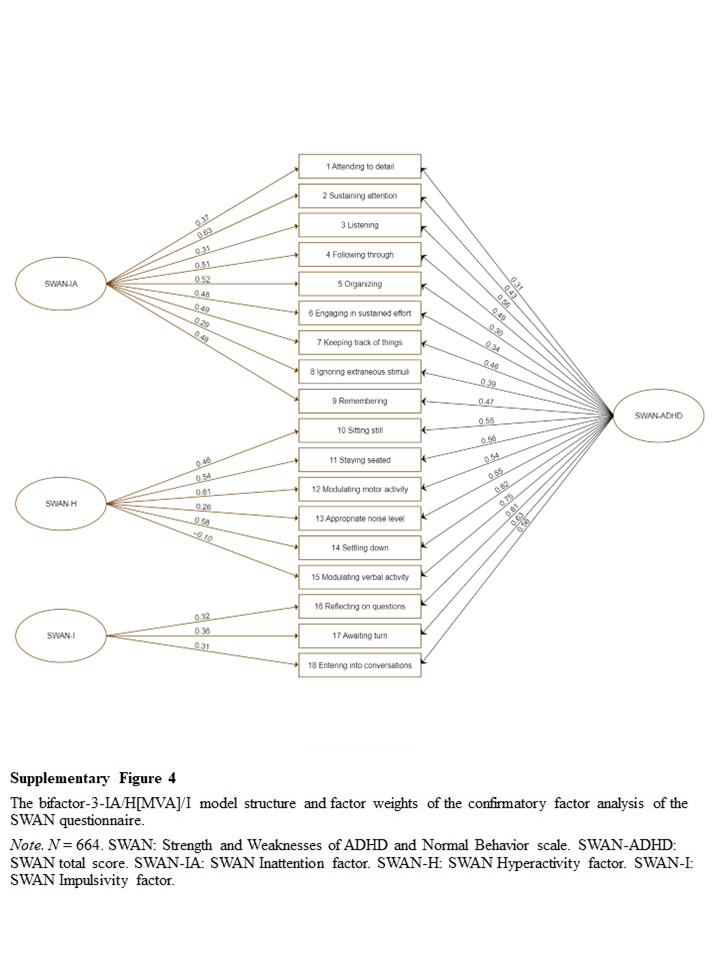

Supplement: Supplementary file 1 [file DataSheet_1.zip › Supplementary Figure 4.TIF]

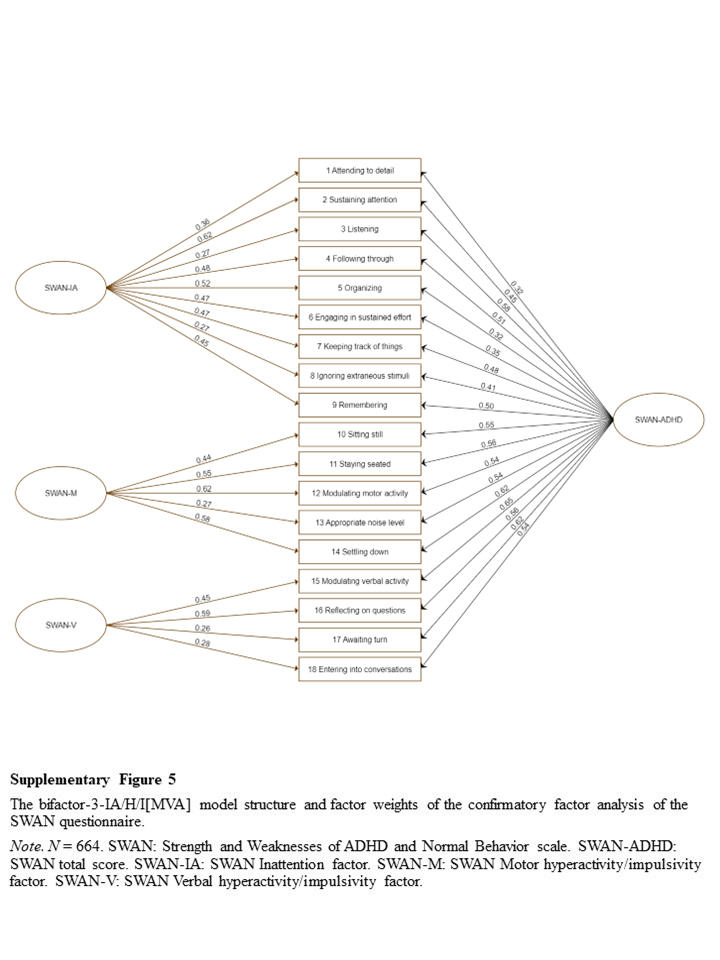

Supplement: Supplementary file 1 [file DataSheet_1.zip › Supplementary Figure 5.TIF]
